# Multimode ultrasonic technique is recommended for the differential diagnosis of thyroid cancer

**DOI:** 10.7717/peerj.9112

**Published:** 2020-05-04

**Authors:** Juan Wang, Xin He, Li Ma, Miao Li, Lei Sun, Jue Jiang, Qi Zhou

**Affiliations:** 1 Department of Ultrasound, The Second Affiliated Hospital, Medical School of Xi’an Jiaotong University, Xi’an, China; 2 Department of Pathology, The Second Affiliated Hospital, Medical School of Xi’an Jiaotong University, Xi’an, China

**Keywords:** Thyroid nodule, B-mode ultrasound, Contrast-enhanced ultrasound, Shear wave elastography

## Abstract

**Background:**

B-mode ultrasound is one of the most commonly used imaging techniques for evaluating thyroid nodules due to its noninvasive property and excellent performance in terms of discriminating between benign and malignant nodules. However, the accuracy of differential diagnosis strongly depends on the experience of ultrasonographers. In addition to B-mode ultrasound, the elastic mode and contrast-enhanced mode have shown complimentary value in the diagnosis of thyroid nodules. The combination of multiple modes in ultrasonic techniques may effectively undermine diagnostic subjectiveness and improve accuracy. In this study, we evaluated the diagnostic value of combining the three ultrasonic modes for differentiating thyroid cancers.

**Methods:**

In this retrospective study, we analyzed a total of 196 thyroid nodules with suspected malignancies from 185 patients who gave informed consent. Xi’an Jiaotong University granted ethical approval (No. 2018200) to carry out the study within its facilities. All the patients received ultrasonic examinations with the B mode, elastic mode and contrast-enhanced mode, followed by histopathological confirmation by fine-need aspiration or surgery. A predictive multivariate logistic regression model was selected to integrate the variety of data obtained from the three modes.

**Results:**

The combination of three ultrasonic techniques for differentiating malignant from benign thyroid nodules showed the highest diagnostic accuracy of 0.985 compared to the B mode alone (0.841) and the two-mode combination. The accuracy of the B mode combined with the elastic technique was 0.954, and the accuracy of the B mode combined with the contrast-enhanced technique was 0.960.

**Discussion:**

Multimode ultrasonic techniques should be recommended to patients with suspected malignant thyroid nodules in routine clinical practice.

## Introduction

Thyroid nodules are a common finding in symptomatic and asymptomatic patients, and they have a malignancy risk rate of approximately 5–15% (Brito et al., 2014). The detection rate of thyroid nodules is dramatically rising due to increased sensitivity in diagnostic imaging tools (Brito, Morris & Montori, 2013; Hoang, Nguyen & Davies, 2015). The current thyroid management guidelines recommend fine-needle aspiration (FNA) for almost all thyroid nodules with suspected malignancies to acquire histopathological confirmation (Haugen et al., 2016; Gharib et al., 2016). However, FNA is invasive and yields nondiagnostic results in approximately 25% of samples (Durante et al., 2018). The high detection rate for thyroid nodules and the relatively low accuracy of diagnosis for thyroid cancer are leading to unnecessary FNA procedures and unwarranted surgeries, which results in economic burden and worsens quality of life. Thus, it is important to improve the accuracy of diagnosis for differentiating thyroid cancer.

B-mode ultrasound is one of the most commonly used imaging techniques for screening thyroid nodules. A diagnosis made by B-mode ultrasound depends on the presence of grayscale features of malignancy and benignity, mainly including size, shape, margin, composition, echogenicity and echogenic foci (Xie et al., 2016). By counting these grayscale features, some risk stratification guidelines have been generated. Of them, the thyroid imaging reporting and data system (TI-RADS) adopted by the American College of Radiology (ACR) is most widely accepted in routine clinical practice. The ACR TI-RADS was constructed from multicenter big data by evaluating the association between five B-mode features and malignancy. The scoring system classifies thyroid nodules into five classes, from TR1 to TR5. Patients with TR3, TR4 and TR5 thyroid nodules are recommended to undergo FNA (Tessler et al., 2017). Although the ACR TI-RADS is robust in terms of statistics, the judgment of the grayscale features of nodules in B-mode ultrasound remains subjective. The diagnostic accuracy heavily depends on the experience of ultrasonographers and the performance of imaging devices (Hoang et al., 2007).

In addition to traditional B-mode ultrasound, ultrasound elasticity and contrast-enhanced techniques have been reported to be valuable in differentiating thyroid cancers (Sui et al., 2016; McQueen & Bhatia, 2015). Shear wave elastography (SWE) is a noninvasive tool that quantitatively reflects the stiffness property of nodules by measuring five elasticity values based on Young’s modulus (Dietrich et al., 2017). In recent years, an increasing number of studies on SWE have shown its good performance in distinguishing malignant thyroid nodules from benign nodules. However, the stability of elastic measurements is easily affected by the swallowing and breathing of subjects, the thyroid location and size, and sonographers’ experience (Veyrieres et al., 2012; Hu, Liu & Qian, 2017; Swan et al., 2019). Contrast-enhanced ultrasound (CEUS) reflects the perfusion of contrast agents within a nodule. After a patient receives an intravenous injection of ultrasonic contrast agent, which consists of gas-filled microbubbles, the contrast agent travels with the blood flow and reaches a targeted thyroid nodule in a few seconds (Lindner, 2004). By switching to the contrast mode of ultrasonic devices, the contrast-enhanced perfusion of agents can be detected differently between benign and malignant thyroid nodules. Previous studies have reported that benign thyroid nodules tend to show homogeneous iso/hyperechogenic contrast enhancement, while malignant nodules tend to show heterogeneous hypoechogenic contrast enhancement (Jiang et al., 2015; Zhang et al., 2010; Ma et al., 2016). However, contrast agents remain within nodules for only a few seconds; thus, the observed results are operator-dependent and easily affected by patients’ swallowing.

In fact, no single ultrasonic technique is perfect. These techniques tend to be complementary to each other. Thus, it may be a promising approach to perform a multimode ultrasonic technique that includes the B mode, elastic mode, and contrast-enhanced mode to improve the accuracy of diagnosis for differentiating thyroid cancer and to decrease unnecessary FNA or unwarranted surgery for benign nodules.

## Materials and Methods

### Patients

In this retrospective study, a total of 196 thyroid nodules from 185 patients were analyzed. Written informed consent was obtained from all the subjects according to the World Medical Association Declaration of Helsinki revised in 2000. Xi’an Jiaotong University granted ethical approval (No. 2018200) to carry out the study within its facilities. All the thyroid nodules were suspected to be malignant and were scored as TR3, TR4 and TR5 nodules according to the ACR TI-RADS (Gharib et al., 2016). Nodules with >25% cystic components were excluded from the study (Zhang et al., 2010). Histopathological examinations after surgical excision or US-guided FNA indicated that of the 196 nodules, 81 (41.33%) were malignant and 115 (58.67%) were benign (Table 1). Of the 185 patients, 151 (81.6%) were males, and 34 were (18.4%) females. The mean age was 45.21 years (ranging from 16 to 84). These patients underwent ultrasound examinations with the B mode, elastic mode and contrast mode sequentially.

**Table 1:** Histopathological types of thyroid nodules.

Histopathological subtype*	Counts (proportion)
Total	196
Benignity	115 (58.67% )
Nodular goiters	87
Follicular adenoma	23
Adenoma	3
Subacute thyroiditis	2
Malignancy	81 (41.33%)
Papillary thyroid carcinoma (PTC)	57
Follicular variant of papillary thyroid carcinoma (FV-PTC)	15
Follicular thyroid carcinoma (FTC)	1
Medullary thyroid carcinoma (MTC)	5
Metastatic carcinoma	2
Undifferentiated carcinoma	1

**Note:**

* The specimens sent for histology were acquired by surgical excision or ultrasound-guided fine-needle aspiration biopsies.

### B-mode examination

B-mode ultrasound examination was performed using an Aixplorer US scanner (Supersonic Imagine, Aix-en-Provence, France) with a 4–15 MHz linear transducer. The patients were instructed to maintain a supine position with their heads backwards. The number, size, shape (ratio of anteroposterior and transverse diameter), margin (irregular/regular), composition (cystic/solid/mixture), echogenicity (hyper/hypo/iso-genicity) and echogenic foci (macro/micro-calcifications) were recorded by a sonographer with 17 years of professional experience in the field. All the measurements were performed at least three times. The mean values were ultimately recorded.

### SWE-mode examination

**Figure 1:**
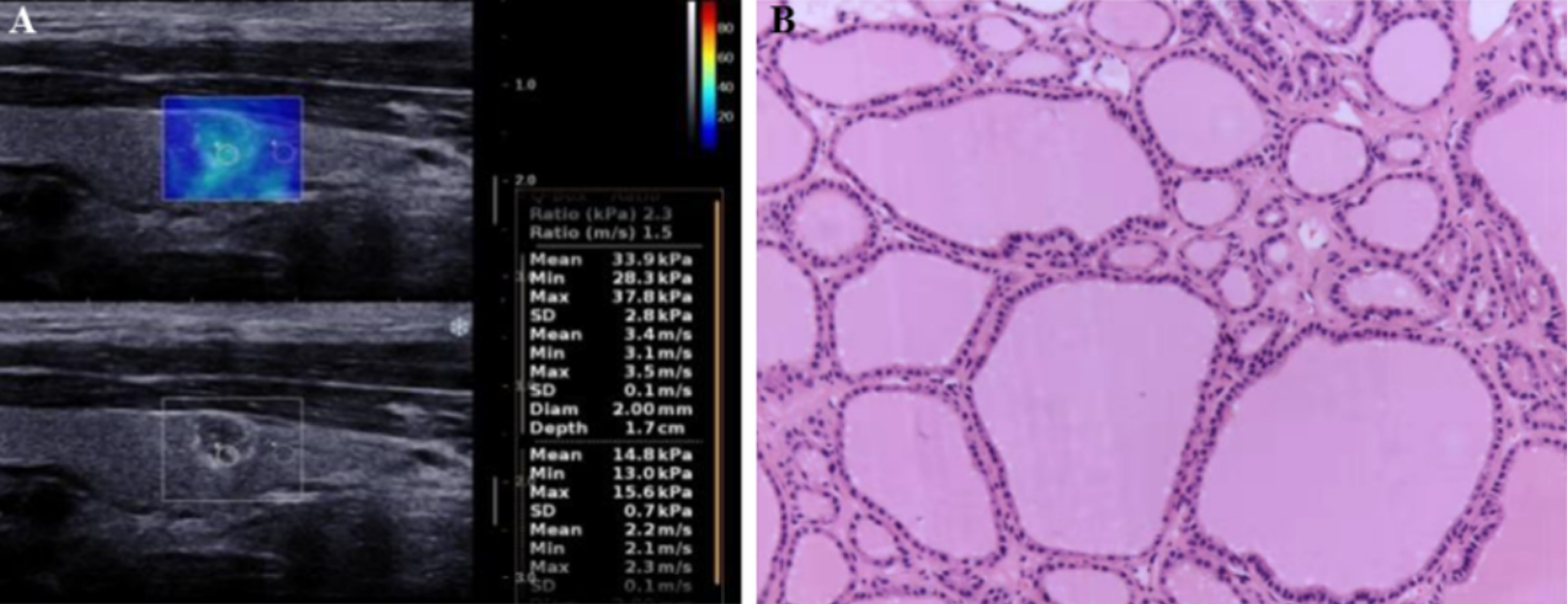
Shear wave elastography and histopathological examination for a benign thyroid nodule in a 43-year-old women. (A) Shear wave elastography. Tissue elasticity distribution represented as a color-coded region (upper) over conventional B-mode image (lower). The right-sided color bar of blue-yellow-red color bar indicates the stiffness of a nodule. (B) Histopathological examination. Hematoxylin and eosin staining for the histological section of the corresponding nodule.

The elastic mode was turned on after a clear grayscale image of thyroid nodules was captured under the B mode. The elastic mode shows both the grayscale image and the SWE image simultaneously (Fig. 1). First, we adjusted the Q-box of interest of the region so that it could cover the whole nodule and the surrounding tissue. Second, we froze the elastic image when the color of the surrounding tissue in the box was almost blue (blue indicates normal soft thyroid tissue). The right-sided color bar in Fig. 1 indicates the stiffness of a nodule, which was set from 0 to 100 kPa. Finally, we measured the stiffness of the nodule tissue as well as its comparative surrounding normal tissue by moving a small circular region where five quantitative elastic Young’s modulus values were displayed on the screen and recorded accordingly (Fig. 1).

### CEUS-mode examination

Thyroid nodules were first visualized clearly under the B mode with a probe with a frequency of 7–14 MHz. Then, the device was turned to the contrast mode. The contrast agent, 2.4 ml of SonoVue (Bracco Inc., Milan, Italy), was injected into the peripheral vein. The patients were instructed to breathe quietly and not to swallow during the inspection. The dynamic contrast-enhanced process was observed and recorded for two minutes. The video data were automatically collected and stored on the machine’s hard disk.

### Statistical analysis

In this study, two types of datasets were analyzed for differentiating malignant from benign thyroid nodules. The qualitative datasets included features of nodules based on the grayscale B mode and types of contrast-enhanced perfusion of nodules and were analyzed with the Chi-square test or Fisher’s exact test (Table 2). The quantitative dataset consisted of elastic indices measured via Young’s modulus property of nodule stiffness based on SWE technology. These data included the E_max, E_min, E_mean, E_SD and E_ratio and were analyzed by Student’s *t*-test (Table S1). A *P* value less than 0.05 was considered statistically significant. In addition, the cutoff value of different elastic parameters, the corresponding sensitivity and specificity, and the area under the receiver operating characteristic (ROC) curve were calculated (Table S2).

**Table 2:** Comparison of qualitative characteristics obtained from the conventional 2D US and CEUS between benign versus malignant thyroid nodules.

Characteristics and classification	Benignity	Malignancy	*P* value
Composition			
Solid or Almost solid	106	80	0.049^b^
Mixed cystic and solid	9	1	
Echogenicity			
Hypo-echoic or very hypo-echoic	90	79	2.68 × 10^−4^^b^
Iso/Mix-echoic	25	2	
Margin			
Irregular or Extra-thyroidal extension	19	43	1.41 × 10^−7^^a^
Regular	96	38	
Shape (A/T)			
>1	14	42	3.76 × 10^−9^^a^
<1	101	39	
Calcification			
Micro-calcification	4	28	2.11 × 10^−8^^a^
Absence or Macro-/Peripheral calcification	111	53	
TI-RADS			
RT3	13	6	0.316^b^
RT4	93	64	
(4,5, and 6 points)	(43, 32, 18)	(2, 8, 54)	
RT5	9	11	
CEUS			
Heterogeneous hypo-enhance	6	71	1.52 × 10^−30^^a^
Homogeneous iso/hyper-enhance	109	10	

**Notes:**

a Chi-square test.

b Fisher exact test.

2D US, two dimensional ultrasound; CEUS, contrast enhance ultrasound; A/T, anteroposterior/transverse diameter; ACR TI-RADS, American college radiologist thyroid imaging report and data system.

**Figure 2:**
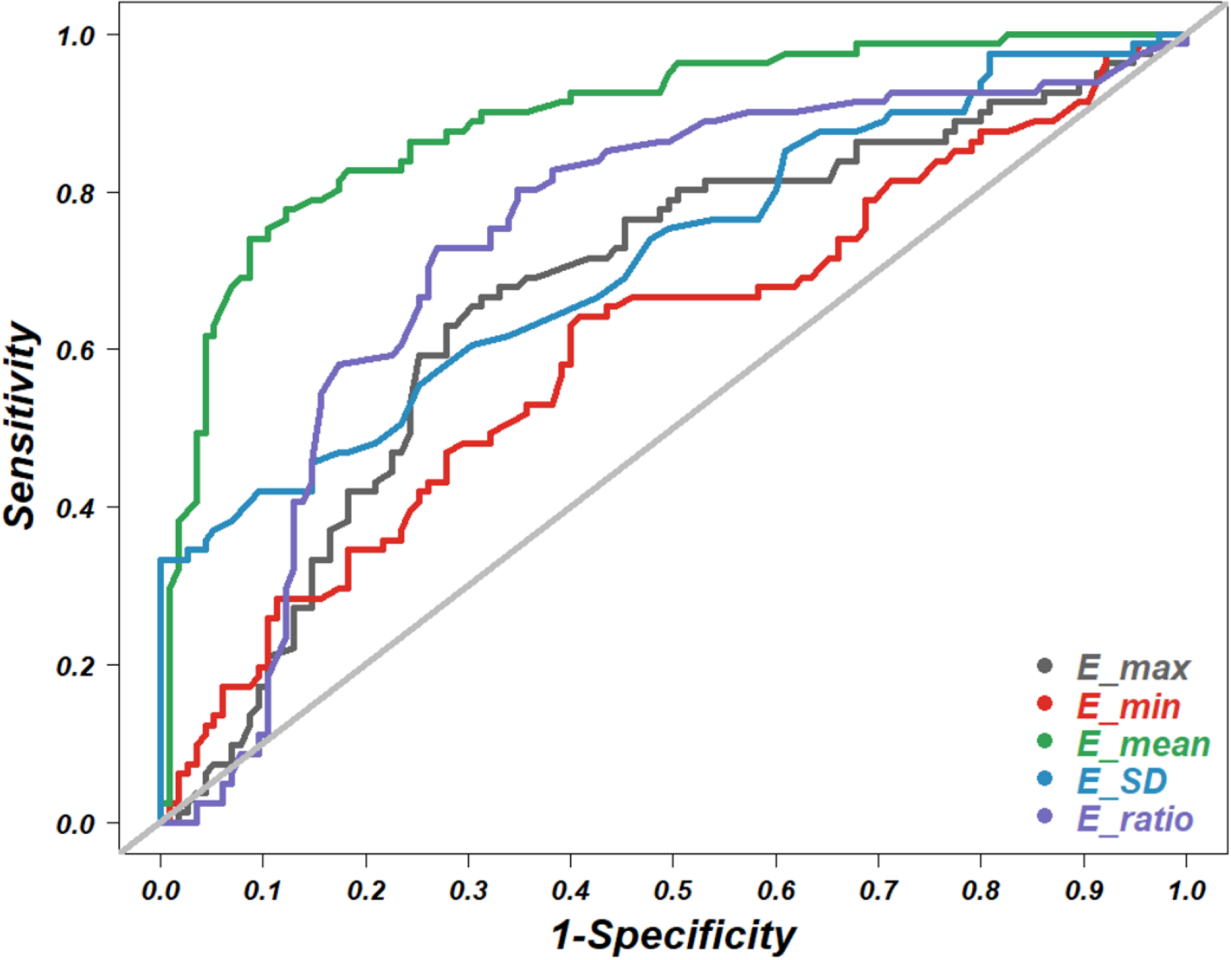
Receiver operating characteristic (ROC) curve. The ROC curves of five elasticity values of shear wave elastography for diagnosis of benign versus malignant thyroid nodules.

**Figure 3:**
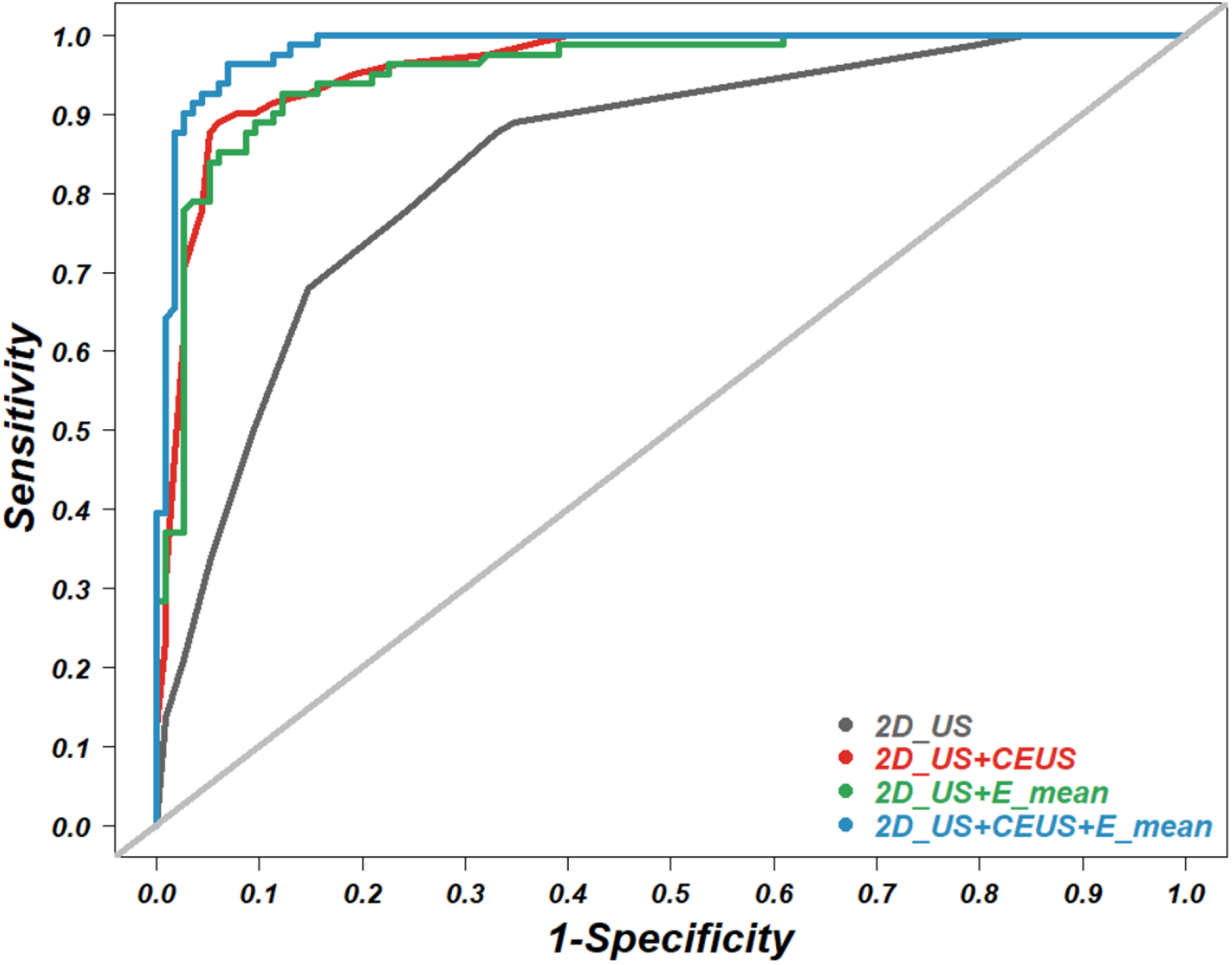
Receiver operating characteristic (ROC) curve. The ROC curves of four combined models for the diagnosis of benign versus malignant thyroid nodules. US, 2D ultrasound; CEUS, contrast enhance ultrasound; SWE, shear wave elastography. E_mean is a SWE parameter.

In this study, multivariate regression analysis was used to predict the risk of malignant thyroid nodules by integrating grayscale features, perfusion types of nodules, and elasticity indices into a comprehensive logistic regression model. Each of five grayscale features, shape, margin, calcification, composition and echogenicity, were analyzed by simple logistic regression. The statistically significant features (*P* < 0.05) and the elasticity indices with the highest accuracy were selected to be entered into the comprehensive logistic regression models (Fig. 2; Tables S3–S7). Finally, the areas under the ROC curves were used for the comparison of diagnostic accuracy among the various comprehensive models (Fig. 3; Table 3). All the raw data for the statistical analyses are included in Table S8. All the statistical analyses were executed with R software version 3.6, and the relevant code is presented in Supplemental Note 1.

**Table 3:** Comparison of ROC curve analysis of multivariate logistic regression models for the prediction of benign versus malignant thyroid nodules.

Combination model	Sensitivity (%)	Specificity (%)	AUC (95% CI)
2D_US	87.65	66.96	0.841 [0.786–0.896]
2D_US+CEUS	88.89	93.91	0.960 [0.935–0.986]
2D_US +E_mean	92.59	87.83	0.954 [0.925–0.982]
2D_US+CEUS+E_mean	96.30	90.04	0.985 [0.971–0.999]

**Note:**

ROC, receiver operating characteristic; AUC, the area under ROC curve; 2D_US, two dimensional ultrasound; CEUS, contrast enhance ultrasound; SWE, shear wave elastography; 95% CI, 95% confidence interval.

## Results

### The diagnostic value of single-mode ultrasonic techniques for differentiating benign from malignant thyroid nodules

To verify the diagnostic performance of the B mode, SWE mode and CEUS mode for differentiating benign from malignant thyroid nodules, we performed relevant statistical analysis of these techniques one by one. The chi-square test or Fisher’s exact test for qualitative datasets with the B mode and CEUS mode showed that all grayscale features, including nodule composition, echogenicity, margin, shape (anteroposterior/transverse diameter), calcification in B mode, and the types of contrast-enhanced effusion in CEUS mode, were significantly different between benign and malignant thyroid nodules (Table 2). The results are consistent with those of previous reports, indicating that all the ultrasonic modes in our study work well individually. Then, we analyzed the effect of grade TR3, TR4 and TR5 nodules calculated with the ACR TI-RADS on the risk stratification. As a result, no difference between benign and malignant thyroid nodules was detected among TR3, TR4 and TR5 nodules (Table 2). The negative result may be due to the small sample size and/or the unstable subjective judgment for grayscale features in B-mode examination.

The quantitative shear wave elastic indices included the E_max, E_min, E_mean, E_SD and E_ratio: their mean ± standard deviation in the malignant nodules were 83.53 ± 31.32, 19.54 ± 6.993, 40.10 ± 7.31, 20.14 ± 6.23 and 3.11 ± 1.30, respectively, while the corresponding indices in the benign nodules were 64.07 ± 32.24, 17.12 ± 36.53, 27.81 ± 7.12, 15.53 ± 3.51 and 2.29 ± 1.68, respectively (Table 1). All the elastic values of the benign nodules were significantly lower than those of the malignant nodules (*P* < 0.05). The accuracy of each elastic value in terms of malignancy prediction was evaluated by ROC curve analysis (Fig. 2). The diagnostic cutoff value and the area under the ROC curve (AUC) are listed in Table 2. The E_mean had the highest diagnostic accuracy, with an AUC of 0.892, and thus was considered to be the most representative SWE index. We added the E_mean to the following multiple logistic regression analysis.

### The diagnostic value of multimode ultrasonic techniques for differentiating benign from malignant thyroid nodules

B-mode, SWE-mode and CEUS-mode ultrasonic techniques are individually effective in differentiating benign from malignant thyroid nodules. The imaging principles of these modes act independently from each other. Thus, it is reasonable to presume that a combination of these modes would show better performance. To estimate the diagnostic value of such a multimode technique, we performed a multivariate logistic regression analysis, which integrated the qualitative grayscale features obtained from the B mode, effusion types obtained from the CEUS mode and the quantitative elastic index E_mean obtained from the SWE mode. Then, we calculated the diagnostic accuracy (AUC) of the B mode alone (2D_US), the B mode combined with the SWE mode (2D_US + E_mean), the B mode combined with the CEUS mode (2D_US + CEUS), and the B mode combined with both modes (2D_US + E_mean + CEUS) (Fig. 3; Table 3). The results indicated that the combination of the three ultrasonic techniques (2D_US + E_mean + CEUS) had the highest accuracy, with an AUC of 0.985, a sensitivity of 96.30% and a specificity of 90.04%. The B mode combined with the CEUS mode showed an AUC of 0.954, which is close to the AUC of 0.960 under the B mode combined with the SWE mode. The B mode alone had the lowest diagnostic accuracy, with an AUC of 0.841. These results suggest that multimode ultrasonic techniques can raise the diagnostic capability of thyroid cancer.

## Discussion

Recently, due to progress in the resolution of ultrasound imaging devices, the identification rate of thyroid nodules has increased to more than 30%. However, only 5–15% of thyroid nodules are malignant (Brito et al., 2014; Frates et al., 2006). The overdiagnosis of “malignant” nodules in ultrasonic screening leads to unnecessary FNA or unwarranted surgery, which results in a poor quality of life for patients. The risk scoring system proposed by the ACR TI-RADS has been widely accepted in clinical routine, where FNA is suggested when TR3, TR4 and TR5 nodules are diagnosed (Tessler et al., 2017). However, the accuracy of the scoring system is easily affected by the experience of ultrasonographers/radiologists due to the overlap of grayscale features in B-mode ultrasound between malignant and benign nodules. This viewpoint is also suggested by our finding that no statistically significant difference in the risk stratification was detected among the suspicious thyroid nodules with grades TR3, TR4 and TR5 (Table 2). Therefore, improving the diagnostic accuracy of suspicious thyroid nodules using multiple noninvasive tools would be meaningful in the current context.

Contrast-enhanced ultrasound has been reported to be helpful in diagnosing thyroid nodules (Jiang et al., 2015; Zhang et al., 2010), although some researchers argue that CEUS is ineffective in differentiating benign from malignant thyroid nodules (Friedrich-Rust et al., 2010). Our previous study showed that CEUS could improve the diagnostic accuracy of differentiating thyroid cancers, suggesting that heterogeneous hypo-echo enhancement (limited agent perfusion within a nodule) may be a high-risk type of malignant thyroid nodule. Such a perfusion type was also adopted in the present study (Jiang et al., 2015). This evidence is opposite to the results of blood perfusion within a breast tumor in which the hyperecho enhancement (more agent perfusion within a nodule) suggests a malignancy (Zhao et al., 2017). This evidence shows that there is a richer blood supply in breast cancer but a much less rich blood supply in thyroid cancer. Additionally, our results showed that the diagnostic accuracy of the B mode combined with the CEUS mode (2D_US+CEUS) was superior to that of the B mode alone (Fig. 3; Table 3), which is consistent with previous reports, providing further evidence for the value of CEUS in the differential diagnosis of thyroid nodules. However, due to the limitation of the short duration of microbubble contrast agents within a thyroid nodule, the judgment of perfusion type is strongly operator-dependent. Additionally, involuntary swallowing in subjects may cause a transient escape of nodules from the scope, thus further increasing the instability of the assessment.

In recent years, the SWE technique, which directly measures the stiffness of nodule tissue, has been increasingly studied for the differential diagnosis of suspicious thyroid nodules. Some have reported that SWE combined with the B mode can help to quantify nodules’ elastic stiffness and increase the accuracy of differentiating thyroid cancer (Veyrieres et al., 2012; Hu, Liu & Qian, 2017; Swan et al., 2019). In our study, of five elasticity values measured by Young’s modulus of nodules, the E_mean showed the highest accuracy, with an AUC of 0.892 (Fig. 2; Table S2). This result suggests that more attention should be paid to the E_mean rather than other values in clinical practice. Some studies claim that the B mode combined with SWE indicates a higher accuracy than the B mode combined with the CEUS technique (Chen et al., 2016). In contrast, our study showed that the B mode combined with the CEUS technique (2D_US + CEUS) had a similar diagnostic capacity to the B mode combined with SWE (2D_US + E_mean) (Fig. 3; Table 3). The inconsistency may be related to the unstable measurements of nodule elasticity, which is easily affected by swallowing and breathing in subjects, thyroid location and size, and sonographers’ experience.

The diagnostic value of the B mode combined with SWE or CEUS in differentiating thyroid cancer has been extensively reported. However, the effects of combining all three modes are rarely studied. Our study indicated that the combination of the three techniques led to the highest accuracy, with an AUC of 0.985, a sensitivity of 98.77% and a specificity of 90.43% (Fig. 3; Table 3). The findings encourage patients with suspicious thyroid nodules diagnosed by the B-mode grayscale ultrasound technique to receive both SWE and CEUS for further confirmation of evidence, which may help to avoid unnecessary FNA and unwarranted surgery of benign nodules. However, considering the instability of the SWE and CEUS techniques in clinical practice, multicenter trials with larger sample sizes would be necessary in future studies.

## Conclusions

No single ultrasonic technique is perfect for differentiating thyroid cancer, and all the techniques tend to be complementary to each other. Additionally, new techniques have been developed, such as the current artificial intelligence, which has been attending to intelligent detection and differential diagnosis of thyroid nodules throughout deep learning methods (Song et al., 2019; Buda et al., 2019) and aims to relieve the labor of sonographers and increase the accuracy of diagnosis. However, the clinical application is still at a very early stage. Since the features of thyroid nodules are very complex and the high quality of images is operator-dependent, machine learning methods are challenging (LeCun, Bengio & Hinton, 2015). Therefore, as knowledge of the benefits and limitations of emerging ultrasonic techniques is continuously expanding, the trend of integrating multiple techniques to diagnose suspicious thyroid nodules will become increasingly apparent. The findings in our study follow this trend and encourage further exploration of the value of multimode ultrasonic techniques in clinical practice.

## Supplemental Information

10.7717/peerj.9112/supp-1 Supplemental Information 1 Comparison of the quantitative elasticity values of SWE between benign versus malignant thyroid nodules. SWE, Share Wave Elastography; E_max, maximum elasticity; E_mean, mean elasticity; E_SD, Standard deviation SWE; E_ratio, ratio of elasticity mean twice.

10.7717/peerj.9112/supp-2 Supplemental Information 2 ROC curve analysis of elasticity values obtained from SWE for differentiating thyroid nodules. SWE, share wave elastography; AUC, the area under a receiver operating characteristic (ROC) curve.

10.7717/peerj.9112/supp-3 Supplemental Information 3 Simple logistic regression of each characteristic of 2D US for the prediction of benign versus malignant thyroid nodules. 2D US: two dimensional ultrasound; B, the estimated logistic coefficient; SE, the standard error of the coefficient; OR, odds ratio; A/T, anteroposterior/transverse diameter.

10.7717/peerj.9112/supp-4 Supplemental Information 4 Multiple logistic regression of the four significant characteristics obtained from 2D US for the prediction of benign versus malignant thyroid nodules. 2D US: two dimensional ultrasound; B, the estimated logistic coefficient; SE, the standard error of the coefficient; OR, odds ratio; A/T, anteroposterior/transverse diameter.

10.7717/peerj.9112/supp-5 Supplemental Information 5 Multiple logistic regression of 2D US combined with CEUS for the prediction of benign versus malignant thyroid nodules. 2D US: two dimensional ultrasound; B, the estimated logit coefficient; SE, the standard error of the coefficient; OR, odds ratio; A/T, anteroposterior/transverse diameter; CEUS, contrast enhance ultrasound.

10.7717/peerj.9112/supp-6 Supplemental Information 6 Multiple logistic regression of 2D US combined with SWE for the prediction of benign versus malignant thyroid nodules. 2D US: two dimensional ultrasound; CEUS, contrast enhance ultrasound; SWE, share wave elastography; B, the estimated logit coefficient; SE, the standard error of the coefficient; OR, odds ratio; A/T, anteroposterior/transverse diameter; E_mean, one elasticity value of SWE.

10.7717/peerj.9112/supp-7 Supplemental Information 7 Multiple logistic regression of 2D US combined with CEUS and SWE for the prediction of benign versus malignant thyroid nodules. 2D US: two dimensional ultrasound; CEUS, contrast enhance ultrasound; SWE, share wave elastography; B, the estimated logistic coefficient; SE, the standard error of the coefficient; OR, odds ratio; A/T: anteroposterior/transverse diameter; E_mean, one elastic value of SWE.

10.7717/peerj.9112/supp-8 Supplemental Information 8 Raw data. A total of 196 thyroid nodules from 185 patients were analyzed in this study.

10.7717/peerj.9112/supp-9 Supplemental Information 9 R code for the statistical analysis.

## Abbreviations

ACR TI-RADS: American College of Radiology Thyroid Image Reporting and Data System
FNA: Fine-needle Aspiration
SWE: Shear Wave Elastography
CEUS: Contrast-Enhanced Ultrasound
2D_US: Two-Dimensional Ultrasound
ROC: Receiver Operating Characteristic
AUC: Area Under the ROC Curve
CI: Confidence Interval
A/T: Anteroposterior/Transverse Diameter
PTC: Papillary Thyroid Carcinoma
FV-PTC: Follicular Variant of Papillary Thyroid Carcinoma
FTC: Follicular Thyroid Carcinoma
MTC: Medullary Thyroid Carcinoma
E_max: Maximum Elasticity
E_mean: Mean Elasticity
E_SD: Standard Deviation SWE
E_ratio: Ratio of Elasticity Mean Twice

## Additional Information and Declarations

### Data Availability

The following information was supplied regarding data availability: The raw measurements are available in Table S8 and Note S1.
