# Supplementary material for: Multimode ultrasonic technique is recommended for the differential diagnosis of thyroid cancer
Source: PeerJ. 2020 May 4;8:e9112. doi: 10.7717/peerj.9112 (PMC7204870; doi:10.7717/peerj.9112)
Supplement: Supplemental Information 1 — SWE, Share Wave Elastography; E_max, maximum elasticity; E_mean, mean elasticity; E_SD, Standard deviation SWE; E_ratio, ratio of elasticity mean twice. [file peerj-08-9112-s001.doc]

**Supplementary table 1.** Comparison of quantitative elasticity values of SWE between benign versus malignant thyroid nodules

| Elasticity values | Pathological finding | Mean ± SD | *P* value ( t-test) |
| --- | --- | --- | --- |
| E_max | Benign | 64.07 ± 32.24 | 3.72x10^-5^ |
|  | Malignant | 83.53 ± 31.32 |  |
| E_min | Benign | 17.12 ± 36.53 | 0.016 |
|  | Malignant | 19.54 ± 6.99 |  |
| E_mean | Benign | 27.81 ± 7.12 | 1.40x10^-23^ |
|  | Malignant | 40.10 ± 7.31 |  |
| E_SD | Benign | 15.53 ± 3.51 | 2.05x10^-8^ |
|  | Malignant | 20.14 ± 6.23 |  |
| E_ratio | Benign | 2.29 ± 1.68 | 1.51x10^-4^ |
|  | Malignant | 3.11 ± 1.30 |  |

SWE, Share Wave Elastography; E_max, maximum elasticity; E_mean, mean elasticity; E_SD, Standard deviation SWE; E_ratio, ratio of elasticity mean twice.
