# Supplementary material for: Multimode ultrasonic technique is recommended for the differential diagnosis of thyroid cancer
Source: PeerJ. 2020 May 4;8:e9112. doi: 10.7717/peerj.9112 (PMC7204870; doi:10.7717/peerj.9112)
Supplement: Supplemental Information 2 — SWE, share wave elastography; AUC, the area under a receiver operating characteristic (ROC) curve. [file peerj-08-9112-s002.doc]

**Supplementary table 2. ROC curve analysis of elasticity values obtained from SWE for differentiating thyroid nodules**

| Elasticity values | Cutoff | Sensitivity (%) | Specificity (%) | AUC (95% CI) |
| --- | --- | --- | --- | --- |
| E_max | 73.75 | 66.67 | 68.70 | 0.673 (0.595-0.751) |
| E_min | 17.25 | 64.20 | 59.13 | 0.607 (0.525-0.688) |
| E_mean | 35.35 | 77.78 | 87.83 | 0.892 (0.846-0.939) |
| E_SD | 19.50 | 33.33 | 100 | 0.717 (0.643-0.791) |
| E_ratio | 2.07 | 80.25 | 65.22 | 0.733 (0.665-0.812) |

SWE, share wave elastography; AUC, the area under a receiver operating characteristic (ROC) curve.
