# Supplementary material for: Multimode ultrasonic technique is recommended for the differential diagnosis of thyroid cancer
Source: PeerJ. 2020 May 4;8:e9112. doi: 10.7717/peerj.9112 (PMC7204870; doi:10.7717/peerj.9112)
Supplement: Supplemental Information 3 — 2D US: two dimensional ultrasound; B, the estimated logistic coefficient; SE, the standard error of the coefficient; OR, odds ratio; A/T, anteroposterior/transverse diameter. [file peerj-08-9112-s003.doc]

**Supplementary table 3. Simple logistic regression of each characteristic of 2D US for the prediction of benign versus malignant thyroid nodules**

| Factor | Intercept | B | SE | Z value | *P* value | OR |
| --- | --- | --- | --- | --- | --- | --- |
| Composition | -4.113 | 1.916 | 1.064 | 1.800 | 0.072 | 6.792 |
| Shape (A/T) | -3.001 | 2.050 | 0.362 | 5.669 | 1.435x 10 ^-8^ | 7.769 |
| Margin | -2.670 | 1.743 | 0.336 | 5.195 | 2.043x 10 ^-7^ | 5.717 |
| Echogenicity | -4.921 | 2.395 | 0.751 | 3.190 | 0.001 | 10.972 |
| Micro-calcification | -2.105 | 1.308 | 0.319 | 4.095 | 4.22x 10 ^-5^ | 3.699 |

2D US: two dimensional ultrasound; B, the estimated logistic coefficient; SE, the standard error of the coefficient; OR, odds ratio; A/T, anteroposterior / transverse diameter.
