# Supplementary material for: Multimode ultrasonic technique is recommended for the differential diagnosis of thyroid cancer
Source: PeerJ. 2020 May 4;8:e9112. doi: 10.7717/peerj.9112 (PMC7204870; doi:10.7717/peerj.9112)
Supplement: Supplemental Information 4 — 2D US: two dimensional ultrasound; B, the estimated logistic coefficient; SE, the standard error of the coefficient; OR, odds ratio; A/T, anteroposterior/transverse diameter. [file peerj-08-9112-s004.doc]

**Supplementary table 4. Multiple logistic regression of the four significant characteristics obtained from 2D US** **for the prediction of benign versus malignant thyroid nodules**

| Factor | B | SE | Z value | *P* value | OR |
| --- | --- | --- | --- | --- | --- |
| Shape (A/T) | 2.110 | 0.414 | 5.099 | 3.414x 10 ^-7^ | 8.252 |
| Margin | 1.267 | 0.391 | 3.240 | 1.197x 10 ^-3^ | 3.638 |
| Echogenicity | 1.648 | 0.793 | 2.078 | 0.038 | 5.196 |
| Micro-calcification | 1.291 | 0.390 | 3.308 | 9.405x 10 ^-4^ | 7.769 |
| Intercept | -9.645 | 1.758 | -5.486 | 4.100x 10 ^-5^ | 6.472 |

2D US: two dimensional ultrasound; B, the estimated logistic coefficient; SE, the standard error of the coefficient; OR, odds ratio; A/T, anteroposterior / transverse diameter.
