# Supplementary material for: Multimode ultrasonic technique is recommended for the differential diagnosis of thyroid cancer
Source: PeerJ. 2020 May 4;8:e9112. doi: 10.7717/peerj.9112 (PMC7204870; doi:10.7717/peerj.9112)
Supplement: Supplemental Information 5 — 2D US: two dimensional ultrasound; B, the estimated logit coefficient; SE, the standard error of the coefficient; OR, odds ratio; A/T, anteroposterior/transverse diameter; CEUS, contrast enhance ultrasound. [file peerj-08-9112-s005.doc]

**Supplementary table 5. Multiple logistic regression of 2D US combined with CEUS** **for the prediction of benign versus malignant thyroid nodules**

| Factor | B | SE | Z value | *P* value | OR |
| --- | --- | --- | --- | --- | --- |
| Shape (A/T) | 1.653 | 0.601 | 2.748 | 6.005x 10 ^-3^ | 5.220 |
| Margin | 0.975 | 0.614 | 1.589 | 0.112 | 2.651 |
| Echogenicity | -0.315 | 0.886 | -0.356 | 0.722 | 0.730 |
| Micro-calcification | 0.857 | 0.582 | 1.474 | 0.140 | 2.357 |
| CEUS | 4.471 | 0.601 | 7.442 | 9.930x 10 ^-14^ | 87.4 |
| Intercept | -10.697 | 1.941 | -5.511 | 3.572x 10 ^-8^ | 2.261 x 10 ^-5^ |

2D US: two dimensional ultrasound; B, the estimated logit coefficient; SE, the standard error of the coefficient; OR, odds ratio; A/T, anteroposterior / transverse diameter; CEUS, contrast enhance ultrasound.
