# Supplementary material for: Multimode ultrasonic technique is recommended for the differential diagnosis of thyroid cancer
Source: PeerJ. 2020 May 4;8:e9112. doi: 10.7717/peerj.9112 (PMC7204870; doi:10.7717/peerj.9112)
Supplement: Supplemental Information 6 — 2D US: two dimensional ultrasound; CEUS, contrast enhance ultrasound; SWE, share wave elastography; B, the estimated logit coefficient; SE, the standard error of the coefficient; OR, odds ratio; A/T, anteroposterior/transverse diameter; E_mean, one elasticity value of SWE. [file peerj-08-9112-s006.doc]

**Supplementary table 6. Multiple logistic regression of 2D US combined with SWE for the prediction of benign versus malignant thyroid nodules**

| Factor | B | SE | Z value | *P* value | OR |
| --- | --- | --- | --- | --- | --- |
| Shape (A/T) | 2.897 | 0.638 | 4.543 | 5.551x 10 ^-6^ | 18.12 |
| Micro-calcification | 1.067 | 0.540 | 1.975 | 0.048 | 22.907 |
| Margin | 1.834 | 0.557 | 3.293 | 9.900x 10 ^-4^ | 6.261 |
| Echogenicity | 0.710 | 1.081 | 0.657 | 0.511 | 2.034 |
| E_mean | 0.259 | 0.043 | 6.004 | 1.921x 10 ^-9^ | 1.296 |
| Intercept | -18.036 | 2.943 | -6.130 | 8.813x 10 ^-10^ | 1.468x 10 ^-8^ |

2D US: two dimensional ultrasound; CEUS, contrast enhance ultrasound; SWE, share wave elastography; B, the estimated logit coefficient; SE, the standard error of the coefficient; OR, odds ratio; A/T, anteroposterior / transverse diameter; E_mean, one elasticity value of SWE.
