# Supplementary material for: Multimode ultrasonic technique is recommended for the differential diagnosis of thyroid cancer
Source: PeerJ. 2020 May 4;8:e9112. doi: 10.7717/peerj.9112 (PMC7204870; doi:10.7717/peerj.9112)
Supplement: Supplemental Information 7 — 2D US: two dimensional ultrasound; CEUS, contrast enhance ultrasound; SWE, share wave elastography; B, the estimated logistic coefficient; SE, the standard error of the coefficient; OR, odds ratio; A/T: anteroposterior/transverse diameter; E_mean, one elastic value of SWE. [file peerj-08-9112-s007.doc]

**Supplementary table 7. Multiple logistic regression of 2D US combined with CEUS and SWE for the prediction of benign versus malignant thyroid nodules**

| Factor | B | SE | Z value | *P* value | OR |
| --- | --- | --- | --- | --- | --- |
| Shape (A/T) | 1.723 | 0.810 | 2.127 | 0.034 | 5.602 |
| Margin | 1.545 | 0.815 | 1.896 | 0.058 | 4.688 |
| Echogenicity | -1.271 | 1.164 | -1.093 | 0.275 | 0.280 |
| Micro-calcification | 0.884 | 0.736 | 1.202 | 0.230 | 2.420 |
| CEUS | 4.400 | 0.814 | 5.406 | 6.448x 10 ^-8^ | 81.474 |
| E_mean | 0.235 | 0.052 | 4.478 | 7.541x 10 ^-6^ | 1.265 |
| Intercept | -17.683 | 3.221 | -5.490 | 4.019x 10 ^-8^ | 2.091x 10 ^-8^ |

2D US: two dimensional ultrasound; CEUS, contrast enhance ultrasound; SWE, share wave elastography; B, the estimated logistic coefficient; SE, the standard error of the coefficient; OR, odds ratio; A/T: anteroposterior / transverse diameter; E_mean, one elastic value of SWE.
