# Supplementary material for: Multimode ultrasonic technique is recommended for the differential diagnosis of thyroid cancer
Source: PeerJ. 2020 May 4;8:e9112. doi: 10.7717/peerj.9112 (PMC7204870; doi:10.7717/peerj.9112)
Supplement: Supplemental Information 9 [file peerj-08-9112-s009.html]

Multi-mode ultrasonic technique is recommended to differential diagnosis of thyroid cancer


# Multi-mode ultrasonic technique is recommended to differential diagnosis of thyroid cancer

#### February 1,2020

####read the data and get the structure of the data

```
origin_data<-read.csv(file="analysis data.csv")
str(origin_data)
```

```
## 'data.frame':    196 obs. of  23 variables:
##  $ sex          : Factor w/ 2 levels "female","male": 1 1 1 1 2 1 1 1 1 1 ...
##  $ age          : int  43 46 49 46 65 36 46 29 56 37 ...
##  $ DiamTrans    : num  7.5 6.6 6.4 6.9 5.8 5.5 6.1 7.9 13.1 6.3 ...
##  $ DiamAP       : num  6.1 4.2 5.8 5.8 5.3 6.4 6.5 8.6 10.3 6 ...
##  $ DiamSag      : num  8.5 8.2 6.3 8.2 6.3 6.7 6.8 8.1 17.9 6.6 ...
##  $ echotexture  : Factor w/ 3 levels "mix","solid",..: 2 2 2 2 2 2 2 2 2 2 ...
##  $ Echogenicity : Factor w/ 4 levels "extre hypo","hypo",..: 2 2 2 2 2 2 2 2 3 2 ...
##  $ Margin       : Factor w/ 2 levels "irregular","regular": 2 2 2 2 2 2 2 1 2 2 ...
##  $ A.T.sag      : Factor w/ 2 levels "<1",">1": 1 1 2 1 1 1 2 2 1 1 ...
##  $ A.T.Trans    : Factor w/ 2 levels "<1",">1": 1 1 2 1 1 2 2 2 1 1 ...
##  $ calcification: Factor w/ 4 levels "absence","circucalcification",..: 1 1 1 1 4 1 1 4 1 4 ...
##  $ Doppler.color: Factor w/ 3 levels "type 1","type 2 ",..: 3 2 3 3 3 3 3 3 2 3 ...
##  $ TI..RADS     : Factor w/ 5 levels "3","4a","4b",..: 3 3 2 2 4 1 4 1 2 1 ...
##  $ SWE100       : Factor w/ 4 levels "colorful","dot-like",..: 2 1 3 1 1 4 3 2 3 2 ...
##  $ MAX          : num  39.8 48 118 65.1 108.9 ...
##  $ MIN          : num  10.5 16.8 28.5 11.9 25.6 18 17.5 10.1 3.4 12 ...
##  $ MEAN         : num  20.7 23.8 35.5 21.4 38.9 40.2 39 38 26.1 47.6 ...
##  $ SD           : num  8 12 20.1 6.3 35.7 35.6 19.1 29.8 17.3 32 ...
##  $ RATIO        : num  3.33 1.8 2.4 1.8 2.9 2.6 3.1 3.2 1.9 2.8 ...
##  $ contrast     : Factor w/ 3 levels "hyper","hypo",..: 3 1 2 3 2 2 2 2 1 2 ...
##  $ Diag.contrast: Factor w/ 3 levels "adenoma","adenomatous  nodular goiters",..: 3 2 1 3 1 1 1 1 2 1 ...
##  $ histology    : Factor w/ 10 levels "adenoma","folli-papillary carcinoma",..: 7 3 8 7 6 2 8 5 3 2 ...
##  $ hist.index   : Factor w/ 2 levels "benign","malignant": 1 1 2 1 2 2 2 2 1 2 ...
```

```
library(hypergea)
```

```
##ACR TI-RADS for risk clasification on 
#echotexture.tab
#echotexture.tab
echotexture.tab<-table(origin_data$hist.index,origin_data$echotexture)
echotexture.tab.1<-cbind(echotexture.tab[,2],echotexture.tab[,1]+echotexture.tab[,3])
colnames(echotexture.tab.1)<-c("solid","mix/sponge")
echotexture.tab.1
```

```
##           solid mix/sponge
## benign      106          9
## malignant    80          1
```

```
expected_echotexture<-getExpectedValues(echotexture.tab.1)
fisher.test(echotexture.tab.1)$p.value
```

```
## [1] 0.04857561
```

```
#echotexture.tab
Echogenicity.tab<-table(origin_data$hist.index,origin_data$Echogenicity )
Echogenicity.tab
```

```
##            
##             extre hypo hypo iso mixtureechogenicity
##   benign             1   89  20                   5
##   malignant          8   71   1                   1
```

```
Echogenicity.tab.1<-cbind(Echogenicity.tab[,1]+Echogenicity.tab[,2],Echogenicity.tab[,3]+Echogenicity.tab[,4])

colnames(Echogenicity.tab.1)<-c("hypo","mix/iso")
Echogenicity.tab.1
```

```
##           hypo mix/iso
## benign      90      25
## malignant   79       2
```

```
getExpectedValues(Echogenicity.tab.1)
```

```
##               hypo  mix/iso
## benign    99.15816 15.84184
## malignant 69.84184 11.15816
```

```
chisq.test(Echogenicity.tab.1)$p.value
```

```
## [1] 0.0002683035
```

```
#Margin
Margin.tab<-table(origin_data$hist.index,origin_data$Margin)
Margin.tab
```

```
##            
##             irregular regular
##   benign           19      96
##   malignant        43      38
```

```
getExpectedValues(Margin.tab)
```

```
##            
##             irregular  regular
##   benign     36.37755 78.62245
##   malignant  25.62245 55.37755
```

```
chisq.test(Margin.tab)$p.value
```

```
## [1] 1.406017e-07
```

```
#A?T
A.T.tab<-table(origin_data$hist.index, origin_data$A.T.Trans)
A.T.tab
```

```
##            
##              <1  >1
##   benign    101  14
##   malignant  39  42
```

```
getExpectedValues(A.T.tab)
```

```
##            
##                   <1       >1
##   benign    82.14286 32.85714
##   malignant 57.85714 23.14286
```

```
chisq.test(A.T.tab)$p.value
```

```
## [1] 3.760412e-09
```

```
#echotexture.tab
Echogenicity.tab<-table(origin_data$hist.index,origin_data$Echogenicity )
Echogenicity.tab
```

```
##            
##             extre hypo hypo iso mixtureechogenicity
##   benign             1   89  20                   5
##   malignant          8   71   1                   1
```

```
Echogenicity.tab.1<-cbind(Echogenicity.tab[,1]+Echogenicity.tab[,2],Echogenicity.tab[,3]+Echogenicity.tab[,4])

colnames(Echogenicity.tab.1)<-c("hypo","mix/iso")
Echogenicity.tab.1
```

```
##           hypo mix/iso
## benign      90      25
## malignant   79       2
```

```
getExpectedValues(Echogenicity.tab.1)
```

```
##               hypo  mix/iso
## benign    99.15816 15.84184
## malignant 69.84184 11.15816
```

```
chisq.test(Echogenicity.tab.1)$p.value
```

```
## [1] 0.0002683035
```

```
#Margin
Margin.tab<-table(origin_data$hist.index,origin_data$Margin)
Margin.tab
```

```
##            
##             irregular regular
##   benign           19      96
##   malignant        43      38
```

```
getExpectedValues(Margin.tab)
```

```
##            
##             irregular  regular
##   benign     36.37755 78.62245
##   malignant  25.62245 55.37755
```

```
chisq.test(Margin.tab)$p.value
```

```
## [1] 1.406017e-07
```

```
#A?T
A.T.tab<-table(origin_data$hist.index, origin_data$A.T.Trans)
A.T.tab
```

```
##            
##              <1  >1
##   benign    101  14
##   malignant  39  42
```

```
getExpectedValues(A.T.tab)
```

```
##            
##                   <1       >1
##   benign    82.14286 32.85714
##   malignant 57.85714 23.14286
```

```
chisq.test(A.T.tab)$p.value
```

```
## [1] 3.760412e-09
```

```
##calcification
calcification.tab<-table(origin_data$hist.index, origin_data$calcification)
calcification.tab.1<-cbind(calcification.tab[,1]+calcification.tab[,2]+calcification.tab[,3],calcification.tab[,4])
calcification.tab.1
```

```
##           [,1] [,2]
## benign     111    4
## malignant   53   28
```

```
getExpectedValues(calcification.tab.1)
```

```
##               [,1]     [,2]
## benign    96.22449 18.77551
## malignant 67.77551 13.22449
```

```
chisq.test(calcification.tab.1)$p.value
```

```
## [1] 2.111949e-08
```

```
#TI..RADS
TI..RADS.tab<-table(origin_data$hist.index, origin_data$TI..RADS)
TI..RADS.tab.1<-cbind(TI..RADS.tab[,1],TI..RADS.tab[,2]+TI..RADS.tab[,3]+TI..RADS.tab[,4],TI..RADS.tab[,5])
getExpectedValues(TI..RADS.tab.1)
```

```
##                [,1]     [,2]      [,3]
## benign    11.147959 92.11735 11.734694
## malignant  7.852041 64.88265  8.265306
```

```
chisq.test(TI..RADS.tab.1)$p.value
```

```
## [1] 0.3155146
```

```
#CEUS
CEUS_data<-table(origin_data$contrast,origin_data$hist.index)
CEUS_data.new<-rbind(CEUS_data[2,],CEUS_data[3,],CEUS_data[1,])
rownames(CEUS_data.new)<-c("Heterogeneous hypo-enhancement","Iso-enhancement","Hyper-enhancement")
CEUS_data.new.1<-rbind(CEUS_data.new[1,],CEUS_data.new[2,]+CEUS_data.new[3,])
rownames(CEUS_data.new.1)<-c("Hetero","homo")

library(hypergea)
getExpectedValues(CEUS_data.new.1)
```

```
##          benign malignant
## Hetero 45.17857  31.82143
## homo   69.82143  49.17857
```

```
chisq.test(CEUS_data.new.1)$p.value
```

```
## [1] 1.515884e-30
```

```
###Make the Roc curve
###SWE
CEUS_res<-origin_data$contrast
CEUS_results<-as.numeric(CEUS_res) #1 indicates iso, 3 indicates hyper, and 2 indicates hypo
CEUS_results[which(CEUS_results==3)]<-1
#CEUS_results[which(CEUS_results==1)]<-3
#CEUS_results[which(CEUS_results==2)]<-1
###ROC
library(pROC)
par(mar=c(4.7,5.2,0.1,0.2))
roc.out<-roc(origin_data$hist.index,CEUS_results)
tmp.roc<-cbind(roc.out$sensitivities,roc.out$specificities,roc.out$thresholds)
roc.out$auc
```

```
## Area under the curve: 0.9122
```

```
tmp.roc[which.max(tmp.roc[,1]+tmp.roc[,2]),]
```

```
## [1] 0.8765432 0.9478261 1.5000000
```

```
plot(1-roc.out$specificities,roc.out$sensitivities,type="l",lwd=3,xlab="",ylab="",xaxt="n",yaxt="n")
axis(1,seq(0,1,by=0.1),cex.axis=1.3,font=4,las=1.5)
axis(2,seq(0,1,by=0.1),cex.axis=1.3,las=2,font=4)
mtext("1-Specificity",side=1,font=4,cex=1.5,line=3)
mtext("Sensitivity",side=2,font=4,cex=1.5,line=3.1)


#####roc.out<-roc(origin_data$hist.index,CEUS_results)
calcif_res<-origin_data$calcification
calcif_results<-as.numeric(calcif_res) #3 indicates type3 , 1 indicates type 1, and 2 indicates type 1.
calcif_results[which(calcif_results==2)]<-2
calcif_results[which(calcif_results==3)]<-2
calcif_results[which(calcif_results==4)]<-2

###ROC
library(pROC)
calcif_roc.out<-roc(origin_data$hist.index,calcif_results)
calcif_tmp.roc<-cbind(calcif_roc.out$sensitivities,calcif_roc.out$specificities,calcif_roc.out$thresholds)
calcif_roc.out$auc
```

```
## Area under the curve: 0.6426
```

```
calcif_tmp.roc[which.max(calcif_tmp.roc[,1]+calcif_tmp.roc[,2]),]
```

```
## [1] 0.4938272 0.7913043 1.5000000
```

```
points(1-calcif_roc.out$specificities,calcif_roc.out$sensitivities,type="l",lwd=3,xlab="",ylab="",xaxt="n",yaxt="n",col=2)


####origin_data$A.T.Trans
###ROC
##roc.out<-roc(origin_data$hist.index,CEUS_results)
AT_res<-origin_data$A.T.Trans
AT_results<-as.numeric(AT_res) #3 indicates type3 , 1 indicates type 1, and 2 indicates type 1.
AT_roc.out<-roc(origin_data$hist.index,AT_results)
AT_tmp.roc<-cbind(AT_roc.out$sensitivities,AT_roc.out$specificities,AT_roc.out$thresholds)
AT_roc.out$auc
```

```
## Area under the curve: 0.6984
```

```
AT_tmp.roc[which.max(AT_tmp.roc[,1]+AT_tmp.roc[,2]),]
```

```
## [1] 0.5185185 0.8782609 1.5000000
```

```
points(1-AT_roc.out$specificities,AT_roc.out$sensitivities,type="l",lwd=3,xlab="",ylab="",xaxt="n",yaxt="n",col=3)


###$ Margin 
Margin_res<-origin_data$Margin
Margin_results<-as.numeric(Margin_res) #3 indicates type3 , 1 indicates type 1, and 2 indicates type 1.
Margin_results[which(Margin_results==1)]<-3
Margin_results[which(Margin_results==2)]<-1
Margin_results[which(Margin_results==3)]<-2


Margin_roc.out<-roc(origin_data$hist.index,Margin_results)
Margin_tmp.roc<-cbind(Margin_roc.out$sensitivities,Margin_roc.out$specificities,Margin_roc.out$thresholds)
Margin_roc.out$auc
```

```
## Area under the curve: 0.6828
```

```
points(1-Margin_roc.out$specificities,Margin_roc.out$sensitivities,type="l",lwd=3,xlab="",ylab="",xaxt="n",yaxt="n",col=4)
#$ echotexture 
echotexture_res<-origin_data$echotexture
echotexture_results<-as.numeric(echotexture_res) #3 indicates type3 , 1 indicates type 1, and 2 indicates type 1.
echotexture_results[which(echotexture_results==3)]<-1

echotexture_roc.out<-roc(origin_data$hist.index,echotexture_results)
echotexture_tmp.roc<-cbind(echotexture_roc.out$sensitivities,echotexture_roc.out$specificities,Margin_roc.out$thresholds)
echotexture_roc.out$auc
```

```
## Area under the curve: 0.533
```

```
echotexture_tmp.roc[which.max(echotexture_tmp.roc[,1]+echotexture_tmp.roc[,2]),]
```

```
## [1] 0.98765432 0.07826087 1.50000000
```

```
points(1-echotexture_roc.out$specificities,echotexture_roc.out$sensitivities,type="l",lwd=3,xlab="",ylab="",xaxt="n",yaxt="n",col=5)


###$ echogenicity 
Echogenicity_res<-origin_data$Echogenicity
Echogenicity_results<-as.numeric(Echogenicity_res) #3 indicates type3 , 1 indicates type 1, and 2 indicates type 1.
Echogenicity_results[which(Echogenicity_results==1)]<-2
Echogenicity_results[which(Echogenicity_results==4)]<-1
Echogenicity_results[which(Echogenicity_results==3)]<-1


Echogenicity_roc.out<-roc(origin_data$hist.index,Echogenicity_results)
Echogenicity_tmp.roc<-cbind(Echogenicity_roc.out$sensitivities,Echogenicity_roc.out$specificities,Echogenicity_roc.out$thresholds)
Echogenicity_roc.out$auc
```

```
## Area under the curve: 0.5963
```

```
Echogenicity_tmp.roc[which.max(Echogenicity_tmp.roc[,1]+Echogenicity_tmp.roc[,2]),]
```

```
## [1] 0.9753086 0.2173913 1.5000000
```

```
points(1-Echogenicity_roc.out$specificities,Echogenicity_roc.out$sensitivities,type="l",lwd=3,xlab="",ylab="",xaxt="n",yaxt="n",col=6)

abline(a=0,b=1,col="gray",lwd=3)
nam<-c("CEUS","Calcification","A/T","Margin","Echotexture","Echogenicity")
legend("bottomright",nam, pch = 19,bty="n",col=1:6,cex=1.7,text.col=1:7,text.font=4)
```

```
###############risik classification
###
His<-as.numeric(origin_data$hist.index)
His[which(His==1)]<-0
His[which(His==2)]<-1
data.news<-data.frame(His=His,calcif=calcif_results,AT=AT_results,CEUS=CEUS_results,
                      Margin=Margin_results,echotexture=echotexture_results,Echogenicity=Echogenicity_results,
                      swe_mean= origin_data$MEAN,age=origin_data$age)
#single logistic regression


#single logistic regression

calcif.lg<-glm(His~calcif,data=data.news,family=binomial)
summary(calcif.lg)$coefficients####ok
```

```
##              Estimate Std. Error  z value     Pr(>|z|)
## (Intercept) -2.105401  0.4562714 -4.61436 3.943081e-06
## calcif       1.308113  0.3194462  4.09494 4.222781e-05
```

```
odds.1<-exp(coef(calcif.lg)[2])
odds.1
```

```
##   calcif 
## 3.699187
```

```
AT.lg<-glm(His~AT,data=data.news,family=binomial)
summary(AT.lg)$coefficients####ok
```

```
##              Estimate Std. Error   z value     Pr(>|z|)
## (Intercept) -3.001730  0.4872431 -6.160642 7.245059e-10
## AT           2.050171  0.3616350  5.669172 1.434893e-08
```

```
odds.2<-exp(coef(AT.lg)[2])
odds.2
```

```
##       AT 
## 7.769231
```

```
##############
Margin.lg<-glm(His~Margin,data=data.news,family=binomial)
summary(Margin.lg)$coefficients####ok
```

```
##              Estimate Std. Error   z value     Pr(>|z|)
## (Intercept) -2.670285  0.4720349 -5.656965 1.540728e-08
## Margin       1.743523  0.3355886  5.195418 2.042607e-07
```

```
odds.3<-exp(coef(Margin.lg)[2])
odds.3
```

```
##   Margin 
## 5.717452
```

```
#################
Echogenicity.lg<-glm(His~Echogenicity,data=data.news,family=binomial)
summary(Echogenicity.lg)$coefficients####ok
```

```
##               Estimate Std. Error   z value     Pr(>|z|)
## (Intercept)  -4.921095  1.4777569 -3.330112 0.0008681116
## Echogenicity  2.395367  0.7508451  3.190228 0.0014216073
```

```
odds.5<-exp(coef(Echogenicity.lg)[2])
odds.5
```

```
## Echogenicity 
##     10.97222
```

```
###########3
echotexture.lg<-glm(His~echotexture,data=data.news,family=binomial)
summary(echotexture.lg)$coefficients####no
```

```
##              Estimate Std. Error   z value   Pr(>|z|)
## (Intercept) -4.113037   2.113207 -1.946348 0.05161298
## echotexture  1.915812   1.064360  1.799966 0.07186593
```

```
odds.6<-exp(coef(echotexture.lg)[2])
odds.6
```

```
## echotexture 
##    6.792453
```

```
###2D_DIMENSION

###significant two-dimentional two-characteristic were selected entered into the model.
out.1<-glm(His~AT+calcif+Margin+Echogenicity,data=data.news,family=binomial)
summary(out.1)
```

```
## 
## Call:
## glm(formula = His ~ AT + calcif + Margin + Echogenicity, family = binomial, 
##     data = data.news)
## 
## Deviance Residuals: 
##     Min       1Q   Median       3Q      Max  
## -2.4648  -0.5845  -0.2654   0.8337   2.0783  
## 
## Coefficients:
##              Estimate Std. Error z value Pr(>|z|)    
## (Intercept)   -9.6454     1.7580  -5.486 4.10e-08 ***
## AT             2.1104     0.4139   5.099 3.41e-07 ***
## calcif         1.2914     0.3904   3.308  0.00094 ***
## Margin         1.2672     0.3912   3.240  0.00120 ** 
## Echogenicity   1.6479     0.7930   2.078  0.03770 *  
## ---
## Signif. codes:  0 '***' 0.001 '**' 0.01 '*' 0.05 '.' 0.1 ' ' 1
## 
## (Dispersion parameter for binomial family taken to be 1)
## 
##     Null deviance: 265.79  on 195  degrees of freedom
## Residual deviance: 187.39  on 191  degrees of freedom
## AIC: 197.39
## 
## Number of Fisher Scoring iterations: 5
```

```
sum.out.1<-summary(out.1)
sum.out.1$ coefficients
```

```
##               Estimate Std. Error   z value     Pr(>|z|)
## (Intercept)  -9.645382  1.7580234 -5.486493 4.099928e-08
## AT            2.110412  0.4138843  5.099038 3.413837e-07
## calcif        1.291382  0.3904107  3.307752 9.404810e-04
## Margin        1.267200  0.3911652  3.239551 1.197179e-03
## Echogenicity  1.647949  0.7929987  2.078124 3.769798e-02
```

```
odds.1<-exp(coef(out.1))
odds.1
```

```
##  (Intercept)           AT       calcif       Margin Echogenicity 
## 6.472375e-05 8.251638e+00 3.637809e+00 3.550895e+00 5.196312e+00
```

```
Two_Dimension_pred<-predict(out.1)

###swe_mean
############################################
###########################################33

###AT+calcif+Margin+Echogenicity+Echotexture+CEUS
out.3<-glm(His~AT+calcif+Margin+Echogenicity+CEUS,data=data.news,family=binomial)
summary(out.3)
```

```
## 
## Call:
## glm(formula = His ~ AT + calcif + Margin + Echogenicity + CEUS, 
##     family = binomial, data = data.news)
## 
## Deviance Residuals: 
##     Min       1Q   Median       3Q      Max  
## -2.7391  -0.3033  -0.2599   0.3222   2.2768  
## 
## Coefficients:
##              Estimate Std. Error z value Pr(>|z|)    
## (Intercept)  -10.6973     1.9412  -5.511 3.57e-08 ***
## AT             1.6525     0.6015   2.748    0.006 ** 
## calcif         0.8574     0.5816   1.474    0.140    
## Margin         0.9751     0.6136   1.589    0.112    
## Echogenicity  -0.3152     0.8863  -0.356    0.722    
## CEUS           4.4713     0.6008   7.442 9.93e-14 ***
## ---
## Signif. codes:  0 '***' 0.001 '**' 0.01 '*' 0.05 '.' 0.1 ' ' 1
## 
## (Dispersion parameter for binomial family taken to be 1)
## 
##     Null deviance: 265.786  on 195  degrees of freedom
## Residual deviance:  97.434  on 190  degrees of freedom
## AIC: 109.43
## 
## Number of Fisher Scoring iterations: 6
```

```
sum.out.3<-summary(out.3)
sum.out.3$ coefficients
```

```
##                 Estimate Std. Error    z value     Pr(>|z|)
## (Intercept)  -10.6973186  1.9411603 -5.5107859 3.572351e-08
## AT             1.6525497  0.6014672  2.7475308 6.004587e-03
## calcif         0.8573977  0.5815553  1.4743186 1.403958e-01
## Margin         0.9750820  0.6136422  1.5890074 1.120587e-01
## Echogenicity  -0.3152056  0.8862881 -0.3556468 7.221051e-01
## CEUS           4.4713109  0.6008343  7.4418372 9.929455e-14
```

```
odds.3<-exp(coef(out.3))
odds.3
```

```
##  (Intercept)           AT       calcif       Margin Echogenicity 
## 2.260547e-05 5.220273e+00 2.357019e+00 2.651385e+00 7.296389e-01 
##         CEUS 
## 8.747132e+01
```

```
CEUS_2D_pred<-predict(out.3)

#######2D_dimension+E_mean
out.4<-glm(His~AT+calcif+Margin+Echogenicity+swe_mean,data=data.news,family=binomial)
summary(out.4)
```

```
## 
## Call:
## glm(formula = His ~ AT + calcif + Margin + Echogenicity + swe_mean, 
##     family = binomial, data = data.news)
## 
## Deviance Residuals: 
##     Min       1Q   Median       3Q      Max  
## -2.8687  -0.3803  -0.1103   0.3125   2.6256  
## 
## Coefficients:
##               Estimate Std. Error z value Pr(>|z|)    
## (Intercept)  -18.03645    2.94254  -6.130 8.81e-10 ***
## AT             2.89710    0.63773   4.543 5.55e-06 ***
## calcif         1.06729    0.54035   1.975  0.04825 *  
## Margin         1.83444    0.55701   3.293  0.00099 ***
## Echogenicity   0.71008    1.08097   0.657  0.51125    
## swe_mean       0.25900    0.04314   6.004 1.92e-09 ***
## ---
## Signif. codes:  0 '***' 0.001 '**' 0.01 '*' 0.05 '.' 0.1 ' ' 1
## 
## (Dispersion parameter for binomial family taken to be 1)
## 
##     Null deviance: 265.79  on 195  degrees of freedom
## Residual deviance: 108.46  on 190  degrees of freedom
## AIC: 120.46
## 
## Number of Fisher Scoring iterations: 6
```

```
sum.out.4<-summary(out.4)
sum.out.4$ coefficients
```

```
##                 Estimate Std. Error    z value     Pr(>|z|)
## (Intercept)  -18.0364515 2.94254153 -6.1295487 8.812871e-10
## AT             2.8970962 0.63773344  4.5428012 5.551159e-06
## calcif         1.0672887 0.54034983  1.9751810 4.824762e-02
## Margin         1.8344397 0.55700858  3.2933779 9.899134e-04
## Echogenicity   0.7100839 1.08097055  0.6568948 5.112486e-01
## swe_mean       0.2590047 0.04313591  6.0043859 1.920574e-09
```

```
odds.4<-exp(coef(out.4))
odds.4
```

```
##  (Intercept)           AT       calcif       Margin Echogenicity 
## 1.468482e-08 1.812145e+01 2.907486e+00 6.261625e+00 2.034162e+00 
##     swe_mean 
## 1.295640e+00
```

```
swe_mean_2D_pred<-predict(out.4)


###2D+CEUS+swe_mean
out.5<-glm(His~AT+calcif+Margin+Echogenicity+CEUS+swe_mean,data=data.news,family=binomial)
summary(out.5)
```

```
## 
## Call:
## glm(formula = His ~ AT + calcif + Margin + Echogenicity + CEUS + 
##     swe_mean, family = binomial, data = data.news)
## 
## Deviance Residuals: 
##      Min        1Q    Median        3Q       Max  
## -2.98633  -0.21161  -0.06626   0.14167   2.14404  
## 
## Coefficients:
##               Estimate Std. Error z value Pr(>|z|)    
## (Intercept)  -17.68302    3.22095  -5.490 4.02e-08 ***
## AT             1.72305    0.81007   2.127   0.0334 *  
## calcif         0.88381    0.73558   1.202   0.2296    
## Margin         1.54494    0.81480   1.896   0.0579 .  
## Echogenicity  -1.27150    1.16384  -1.093   0.2746    
## CEUS           4.40029    0.81398   5.406 6.45e-08 ***
## swe_mean       0.23481    0.05244   4.478 7.54e-06 ***
## ---
## Signif. codes:  0 '***' 0.001 '**' 0.01 '*' 0.05 '.' 0.1 ' ' 1
## 
## (Dispersion parameter for binomial family taken to be 1)
## 
##     Null deviance: 265.786  on 195  degrees of freedom
## Residual deviance:  61.576  on 189  degrees of freedom
## AIC: 75.576
## 
## Number of Fisher Scoring iterations: 7
```

```
sum.out.5<-summary(out.5)
sum.out.5$ coefficients
```

```
##                 Estimate Std. Error   z value     Pr(>|z|)
## (Intercept)  -17.6830207 3.22095399 -5.489995 4.019455e-08
## AT             1.7230514 0.81007056  2.127039 3.341687e-02
## calcif         0.8838079 0.73557584  1.201518 2.295502e-01
## Margin         1.5449435 0.81479528  1.896112 5.794518e-02
## Echogenicity  -1.2715002 1.16384091 -1.092503 2.746119e-01
## CEUS           4.4002894 0.81397777  5.405909 6.448067e-08
## swe_mean       0.2348076 0.05243805  4.477809 7.541298e-06
```

```
odds.5<-exp(coef(out.5))
odds.5
```

```
##  (Intercept)           AT       calcif       Margin Echogenicity 
## 2.091037e-08 5.601595e+00 2.420098e+00 4.687707e+00 2.804106e-01 
##         CEUS     swe_mean 
## 8.147445e+01 1.264665e+00
```

```
total_pred<-predict(out.5)


####expectation
###ROC
library(pROC)
par(mar=c(4.7,5.2,0.1,0.2))
####
roc.out<-roc(origin_data$hist.index,Two_Dimension_pred)
tmp.roc<-cbind(roc.out[[2]],roc.out[[3]],roc.out[[4]])
roc.out$auc
```

```
## Area under the curve: 0.8408
```

```
ci(roc.out)
```

```
## 95% CI: 0.7859-0.8956 (DeLong)
```

```
tmp.roc[which.max(tmp.roc[,1]+tmp.roc[,2]),]
```

```
## [1]  0.8765432  0.6695652 -0.8156598
```

```
plot(1-roc.out[[3]],roc.out[[2]],type="l",lwd=3,xlab="",ylab="",xaxt="n",yaxt="n")
axis(1,seq(0,1,by=0.1),cex.axis=1.3,font=4,las=1.5)
axis(2,seq(0,1,by=0.1),cex.axis=1.3,las=2,font=4)
mtext("1-Specificity",side=1,font=4,cex=1.5,line=3)
mtext("Sensitivity",side=2,font=4,cex=1.5,line=3.1)


roc.out.4<-roc(origin_data$hist.index, CEUS_2D_pred)
tmp.roc.4<-cbind(roc.out.4[[2]],roc.out.4[[3]],roc.out.4[[4]])
roc.out.4$auc
```

```
## Area under the curve: 0.9604
```

```
ci(roc.out.4)
```

```
## 95% CI: 0.9352-0.9856 (DeLong)
```

```
tmp.roc.4[which.max(tmp.roc.4[,1]+tmp.roc.4[,2]),]
```

```
## [1]  0.8888889  0.9391304 -0.3150589
```

```
points(1-roc.out.4[[3]],roc.out.4[[2]],type="l",lwd=3,xlab="",ylab="",xaxt="n",yaxt="n",col=2)


################
roc.out.5<-roc(origin_data$hist.index, swe_mean_2D_pred)
tmp.roc.5<-cbind(roc.out.5[[2]],roc.out.5[[3]],roc.out.5[[4]])
roc.out.5$auc
```

```
## Area under the curve: 0.9536
```

```
ci(roc.out.5)
```

```
## 95% CI: 0.9251-0.9821 (DeLong)
```

```
tmp.roc.5[which.max(tmp.roc.5[,1]+tmp.roc.5[,2]),]
```

```
## [1]  0.9259259  0.8782609 -0.5738248
```

```
points(1-roc.out.5[[3]],roc.out.5[[2]],type="l",lwd=3,xlab="",ylab="",xaxt="n",yaxt="n",col=3)

##############
roc.out.6<-roc(origin_data$hist.index, total_pred)
tmp.roc.6<-cbind(roc.out.6[[2]],roc.out.6[[3]],roc.out.6[[4]])
roc.out.6$auc
```

```
## Area under the curve: 0.9848
```

```
ci(roc.out.6)
```

```
## 95% CI: 0.9711-0.9985 (DeLong)
```

```
tmp.roc.6[which.max(tmp.roc.6[,1]+tmp.roc.6[,2]),]
```

```
## [1]  0.9629630  0.9304348 -1.2081343
```

```
points(1-roc.out.6[[3]],roc.out.6[[2]],type="l",lwd=3,xlab="",ylab="",xaxt="n",yaxt="n",col=4)

nam<-c("2D_US","2D_US+CEUS","2D_US+E_mean","2D_US+CEUS+E_mean")
legend("bottomright",nam, pch = 19,bty="n",col=1:4,cex=1.5,text.font=4,text.col=1:4)
abline(a=0,b=1,col="gray",lwd=3)
```

```
###################


###################################33
#########################################
```

```
SWE.data<-origin_data[,14:19]
ben.index<-which(origin_data$hist.index=="benign")
mal.index<-which(origin_data$hist.index=="malignant")
malignant.swe<-SWE.data[mal.index,]
benign.swe<-SWE.data[ben.index,]
dim(malignant.swe)
```

```
## [1] 81  6
```

```
dim(benign.swe)
```

```
## [1] 115   6
```

```
apply(malignant.swe[,-1],2,mean)
```

```
##       MAX       MIN      MEAN        SD     RATIO 
## 83.530617 19.535926 40.099259 20.139506  3.112346
```

```
apply(malignant.swe[,-1],2,sd)
```

```
##       MAX       MIN      MEAN        SD     RATIO 
## 31.312350  6.991856  7.313953  6.230864  1.307480
```

```
apply(benign.swe[,-1],2,mean)
```

```
##       MAX       MIN      MEAN        SD     RATIO 
## 64.067652 17.123130 27.810000 15.526087  2.287478
```

```
apply(benign.swe[,-1],2,sd)
```

```
##       MAX       MIN      MEAN        SD     RATIO 
## 32.240533  6.528600  7.124941  3.505036  1.675385
```

```
t.test(malignant.swe$MAX,benign.swe$MAX)$p.value
```

```
## [1] 3.716122e-05
```

```
t.test(malignant.swe$MIN,benign.swe$MIN)$p.value
```

```
## [1] 0.01555529
```

```
t.test(malignant.swe$MEAN,benign.swe$MEAN)$p.value
```

```
## [1] 1.40152e-23
```

```
t.test(malignant.swe$SD,benign.swe$SD)$p.value
```

```
## [1] 2.051814e-08
```

```
t.test(malignant.swe$RATIO,benign.swe$RATIO)$p.value
```

```
## [1] 0.0001510718
```

```
boxplot(benign.swe$MAX,malignant.swe$MAX,names=c("Benign","Malignant"),main="E_max",
        col=(c("gold","darkgreen")),cex.main=1.8,cex.axis=1.5,boxlwd = 2,pch=19)
```

```
boxplot(benign.swe$MIN,malignant.swe$MIN,names=c("Benign","Malignant"),main="E_min",
        col=(c("gold","darkgreen")),cex.main=1.8,cex.axis=1.5,boxlwd = 2,pch=19)
```

```
boxplot(benign.swe$MEAN,malignant.swe$MEAN,names=c("Benign","Malignant"),main="E_mean",
        col=(c("gold","darkgreen")),cex.main=1.8,cex.axis=1.5,boxlwd = 2,pch=19)
```

```
boxplot(benign.swe$MEAN,malignant.swe$MEAN,names=c("Benign","Malignant"),main="SD",
        col=(c("gold","darkgreen")),cex.main=1.8,cex.axis=1.5,boxlwd = 2,pch=19)
```

```
boxplot(benign.swe$RATIO,malignant.swe$RATIO,names=c("Benign","Malignant"),main="E_ratio",
        col=(c("gold","darkgreen")),cex.main=1.8,cex.axis=1.5,boxlwd = 2,pch=19)
```

```
###ROC
library(pROC)
par(mar=c(4.7,5.2,0.1,0.2))
roc.out<-roc(origin_data$hist.index,SWE.data$MAX,ci=TRUE)
tmp.roc<-cbind(roc.out[[2]],roc.out[[3]],roc.out[[4]])
roc.out$auc
```

```
## Area under the curve: 0.6734
```

```
ci(roc.out)
```

```
## 95% CI: 0.5954-0.7513 (DeLong)
```

```
tmp.roc[which.max(tmp.roc[,1]+tmp.roc[,2]),]
```

```
## [1]  0.6666667  0.6869565 73.7500000
```

```
plot(1-roc.out[[3]],roc.out[[2]],type="l",lwd=3,xlab="",ylab="",xaxt="n",yaxt="n")
axis(1,seq(0,1,by=0.1),cex.axis=1.3,font=4,las=1.5)
axis(2,cex.axis=1.3,las=2,font=4)
mtext("1-Specificity",side=1,font=4,cex=1.5,line=3)
mtext("Sensitivity",side=2,font=4,cex=1.5,line=3.3)


roc.out<-roc(origin_data$hist.index,SWE.data$MIN)
roc.out$auc
```

```
## Area under the curve: 0.6068
```

```
ci(roc.out)
```

```
## 95% CI: 0.5252-0.6883 (DeLong)
```

```
tmp.roc<-cbind(roc.out[[2]],roc.out[[3]],roc.out[[4]])
tmp.roc[which.max(tmp.roc[,1]+tmp.roc[,2]),]
```

```
## [1]  0.6419753  0.5913043 17.2500000
```

```
points(1-roc.out[[3]],roc.out[[2]],type="l",lwd=3,col=2)


roc.out<-roc(origin_data$hist.index,SWE.data$MEAN)
roc.out$auc
```

```
## Area under the curve: 0.8924
```

```
ci(roc.out)
```

```
## 95% CI: 0.8461-0.9386 (DeLong)
```

```
tmp.roc<-cbind(roc.out[[2]],roc.out[[3]],roc.out[[4]])
tmp.roc[which.max(tmp.roc[,1]+tmp.roc[,2]),]
```

```
## [1]  0.7777778  0.8782609 35.3000000
```

```
points(1-roc.out[[3]],roc.out[[2]],type="l",lwd=3,col=3)


roc.out<-roc(origin_data$hist.index,SWE.data$SD)
roc.out$auc
```

```
## Area under the curve: 0.7171
```

```
ci(roc.out)
```

```
## 95% CI: 0.6432-0.791 (DeLong)
```

```
tmp.roc<-cbind(roc.out[[2]],roc.out[[3]],roc.out[[4]])
tmp.roc[which.max(tmp.roc[,1]+tmp.roc[,2]),]
```

```
## [1]  0.3333333  1.0000000 19.5000000
```

```
points(1-roc.out[[3]],roc.out[[2]],type="l",lwd=3,col=4)


roc.out<-roc(origin_data$hist.index,SWE.data$RATIO)
roc.out$auc
```

```
## Area under the curve: 0.7382
```

```
ci(roc.out)
```

```
## 95% CI: 0.6647-0.8116 (DeLong)
```

```
tmp.roc<-cbind(roc.out[[2]],roc.out[[3]],roc.out[[4]])
tmp.roc[which.max(tmp.roc[,1]+tmp.roc[,2]),]
```

```
## [1] 0.7283951 0.7304348 2.5900000
```

```
points(1-roc.out[[3]],roc.out[[2]],type="l",lwd=3,col=5)


nam<-c("E_max","E_min","E_mean","E_SD","E_ratio")

abline(a=0,b=1,col="gray",lwd=3)

legend("bottomright",nam, pch = 19,bty="n",col=1:6,cex=1.7,text.col=1:7,text.font=4)
```
